# Performance Enhancement of Polyurethane Acrylate Resin by Urushiol: Rheological and Kinetic Studies

**DOI:** 10.3390/polym16192716

**Published:** 2024-09-25

**Authors:** Yuchi Zhang, Run Fang, Hanyu Xue, Yuansong Ye, Li Chen, Jianrong Xia

**Affiliations:** 1 College of Material and Chemical Engineering, Minjiang University, Fuzhou 350108, China; zyc@mju.edu.cn (Y.Z.); 1763@mju.edu.cn (R.F.); 2280@mju.edu.cn (H.X.); yeyuansong@mju.edu.cn (Y.Y.); 3213103103@stu.mju.edu.cn (L.C.); 2 Fujian Engineering and Research Center of New Chinese Lacquer Materials, Fuzhou 350108, China

**Keywords:** urushiol, non-isothermal, rheological behavior, cure kinetics, rheometer

## Abstract

A natural extract, i.e., urushiol, was employed to effectively cross-link and modify commercial wet-cured polyurethane acrylic resin. Comprehensive characterization of the paint film was performed using techniques such as FTIR, SEM, and TGA. The results indicated that the incorporation of urushiol significantly increased the cross-linking density of the resin, which in turn enhanced the film-forming properties, mechanical strength, and thermal stability of the paint film. Additionally, the study discovered that under isothermal conditions, the dynamic moduli (G′ and G″) of the paint film are related to the gel point frequency by a power law, aligning with the predictions of percolation theory. The application of the autocatalytic model has provided a novel approach to studying non-isothermal kinetic reactions, offering valuable insights for process optimization and further development of urushiol-based polyurethane.

## 1. Introduction

Low-Volatile Organic Compound (VOC) coatings are emerging as a new trend in the paint industry [1,2]. Polyurethane acrylate, denoted as PUA, is a moisture-curing resin with excellent film performance, featuring acrylate and isocyanate functional groups in its molecular structure, which combines the advantages of both polyurethane (PU) and acrylate coatings. The curing process of PUA coatings involves a reaction between excess isocyanate groups and water, making them energy-efficient, environmentally friendly, and virtually free of VOC emissions. Moreover, PUA has been widely used in industrial fields such as eyeglass frames, carbon-fiber and glass-fiber coating, anti-corrosion coatings, automotive, and aviation [3,4,5].

PUA resins have certain drawbacks in industrial applications, including insufficient hardness, slow curing process, long surface drying time, and poor heat resistance. These issues may lead to the detachment of coating film and poor thermal stability, thus limiting its scope of use.

**Figure 1:**
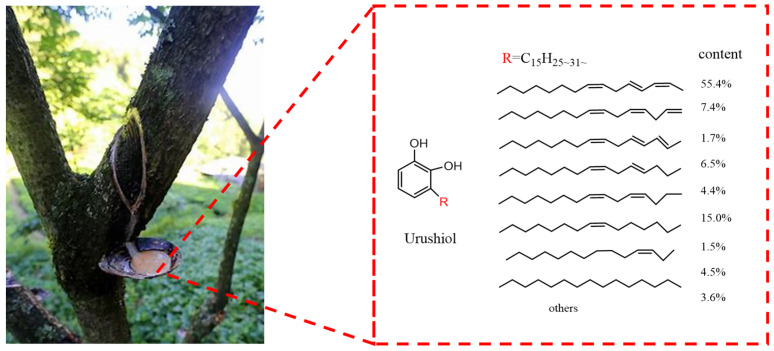
Chemical structure and composition of urushiol.

Raw lacquer is a unique high-quality natural resource exclusive to China, known for its exceptional adhesion, film-forming properties, and eco-friendliness. The main component of raw lacquer, urushiol, is a catechol derivative with an unsaturated long-side chain structure ranging from C15 to C17 [6,7,8], as depicted in Figure 1 [9]. The structural features endow shellac with excellent elasticity, hardness, and durability, which are of significant application value to the coatings industry. As a result of the presence of hydroxyl groups and unsaturated bonds in shellac, it can not only replace polyols to react with isocyanate groups in PUA but also cause cross-linking reactions in PUA to form a three-dimensional network structure, thereby improving the physical and mechanical properties as well as the heat resistance of PUA. Moreover, the modification of acrylate-polyurethane materials with biobased shellac is also a major trend in functional coatings [1].

The curing mechanism is pivotal in determining the behavior and kinetics of curing. Therefore, the study of curing kinetics is of vital importance. It not only enhances our understanding of the curing behavior but also offers theoretical grounding for the interplay between the structure, properties, and processing of resins [10]. For example, Domínguez and his colleagues utilized chemorheology to study the curing kinetics of polyfurfuryl alcohol resin, offering the theoretical foundation for its application in the processing of composite materials [11]. Moreover, rheological dynamics research aids in a better understanding of the chemical reaction processes in thermosetting materials. Wei et al. used non-isothermal rheology to study the curing kinetics of guanidinyldiazobutrol (GAP) spherical propellants and established a kinetic equation, thus confirming that non-isothermal rheology can be applied to study the mechanisms and kinetics of thermosetting reactions [12].

It is well-known that differential scanning calorimetry (DSC) has significant advantages in studying curing kinetics, but it cannot display the evolution of changes in the physical state of the reaction system. Rheological methods can overcome the limitations of DSC technology and exhibit a higher sensitivity to the rheological properties throughout the entire curing process [13,14]. Rheology can identify the critical transitions during resin curing, such as glass transition and gelation, and study the formation of networks through physical and chemical means. Therefore, rheological methods provide a more effective way to study the curing process. These techniques are crucial not only for optimizing processing cycles but also for gaining a fundamental understanding of the relationship between kinetics and mechanical behavior.

In our previous work, rheological methods have been used to study the curing kinetics of shellac/IPDI and shellac/MDI, with experimental data that fit well with the empirical model data [15,16]. This article primarily investigates the isothermal and non-isothermal curing characteristics of the urushiol-modified PUA resin and establishes the rheokinetic curing rules for urushiol-modified PUA resin, and analyzes the impact of the urushiol content on the mechanical properties of the coating, including hardness, flexibility, adhesion, impact resistance and chemical resistance. Additionally, it provides a comprehensive evaluation of the coating’s thermal stability.

## 2. Experimental Section

### 2.1. Materials

Raw lacquer was purchased from the Xi’an Raw Lacquer Coating Research Institute, and 94 wt% urushiol was obtained after extraction with ethanol [9], dried over anhydrous CaCl_2_ before use. The value of the reactive hydroxyl group of urushiol was determined to be 6.7 × 10^−3^ mol/g, measured by the acetylation method according to AOAC official method 965.32 [17,18]. The commercial polyurethane acrylate (PUA) was purchased from Jiangsu Sanmu Group Co., Ltd. (Wuxi, Jiangsu, China), used without further purification, and its parameters are shown in Table 1.

### 2.2. Preparation of the Urushiol Cross-Linking-Modified PUA

A series of urushiol-modified PUA resins was synthesized in the laboratory by changing the proportion of urushiol. The synthesis process is as follows: Under nitrogen atmosphere, 100 g of commercial PUA resin was added to a 250 mL three-necked flask, followed by the addition of 25 g of urushiol. The molar ratio of phenolic hydroxyl to NCO was maintained at around 1:1. The resulting mixture was magnetically stirred for 5 min at 25 °C and subsequently purified using vacuum distillation to yield PUA-U25 resin. By varying the content of urushiol and replicating the synthesis procedure, PUA, PUA-U10, PUA-U15, PUA-U20, and PUA-U30 resins were fabricated, with the mass ratio of urushiol to PUA resin documented in Table 2.

### 2.3. Preparation of the Urushiol Cross-Linking-Modified PUA Coating Films

In the experiment, 25 mm × 75 mm glass slides and 50 mm × 100 mm tinplate pieces were ultrasonically cleaned with acetone, ethanol, and deionized water, followed by drying with nitrogen. Subsequently, the urushiol-modified PUA resins were coated uniformly with a thickness of 100 μm according to the national standard GB/T 1727-1992, and then cured in a 25 °C dryer to avoid humidity effects.

### 2.4. Measurements

Attenuated total reflectance Fourier transform infrared spectroscopy (ATR-FTIR) was recorded on an IS 5 spectrometer produced by Thermo Fisher in the United States with a single reflection diamond crystal ATR unit, using a resolution of 4 cm^−1^. The spectrum of each sample was obtained by averaging 32 scans in a scanning range between 4000 and 400 cm^−1^.

The microstructure of the paint film was observed using a ZEISS EVO MA 15 scanning electron microscope (SEM) at a 5 kV accelerating voltage. For convenience of observation, the samples were sputtered for 30 s using a sputtering gold-plating machine.

The thermal stability of the paint film was tested using a Netzsch STA 449 F3 thermogravimetric analyzer (TG). The test conditions were as follows: from room temperature to 600 °C, with a constant nitrogen flow rate, and a heating rate of 10 °C/min.

### 2.5. Mechanical Properties and Chemical Resistance of the Coating

Glossiness was measured according to the GB/T 8807-1988 standard [19], using a glossiness meter (JFL-B60°, Tianjin jinfulun Technology Co., Ltd., Tianjing, China) to reflect the ratio of light reflected from the surface compared to a standard panel. 

The hardness test was conducted according to the GB/T 6739-2006 standard [20], using a pencil hardness tester (QHQ-A, Tianjin Jingkelian Material Testing Machine Co., Ltd., Tianjin, China). The hardness level ranges from 6B (softest) to 6H (hardest), indicating a gradual increase in hardness.

The adhesion test follows the GB/T 9286-2021 standard [21] and a cross-cutting test was conducted using 100 grid knives to evaluate the adhesion of the coating film. The adhesion level ranges from 0 (best) to 5 (worst).

The bending test (QTY-32, Tianjin Jingkelian Material Testing Machine Co., Ltd., Tianjin, China) was used to determine the elasticity of the coatings, the standard being GB/T 6742-2007 [22]. The coated tinplate was bent along cylindrical axes of different diameters. The minimum bending diameter of the cylindrical shaft was recorded and there were no cracks in the coating along this radius.

The impact resistance test was conducted using a coating film impactor (QCJ-50, Tianjin Jingkelian Material Testing Machine Co., Ltd., Tianjin, China) according to the GB/T 1732-1993 standard [23]. At the maximum impact distance, the coating did not crack. 

The chemical resistance of the coating film was evaluated according to national standards, using acid-base and immersion methods. The dried coated glass panels were immersed in solutions of 10% NaCl, 10% H_2_SO_4_, 10% NaOH, and ethanol (C_2_H_5_OH) at a volume fraction of 95% for a period of 7 days. The adhesion of the coated panels was assessed and any wrinkling or blistering was visually inspected.

### 2.6. Rheological Analysis

In the experiment, a HAAKE Mars 60 rheometer was used for dynamic rheological measurements. The samples were heated by a forced convection furnace with a temperature stability of ±0.1 °C. During the testing process, disposable aluminum parallel plates with a diameter of 25 mm and a gap of 1 mm were used. The steps of the rheological test in the linear viscoelastic region are as follows: (1) Time sweeps were performed at different constant temperatures (40–80 °C) and a constant shear frequency (ω = 6.28 rad/s) in the linear viscoelastic state (strain amplitude 1%) to determine the influence of the gelation process on the characteristic viscoelastic functions (G′, G″, and η*). (2) Frequency sweeps were performed at a fixed temperature (70 °C) in different linear viscoelastic states (with a strain amplitude of 1%) to obtain the viscoelastic characteristic functions (G′, G″, and tanδ) over a wide range of frequencies and times. (3) Non-isothermal curing experiments were conducted in the temperature range of 30 °C to 180 °C with heating rates of 1, 1.5, 2, 3, 4, and 5 °C/min, at a frequency of ω = 6.28 rad/s, and a strain of 1%. These measurement results enable us to verify the validity of the critical exponents based on percolation theory, which are expressed in a power-law form for G′, G″.

## 3. Results and Discussion

### 3.1. Performance Analysis of the Urushiol-Modified PUA

#### 3.1.1. FTIR Characterization of the PUA and Urushiol-Modified PUA

**Figure 2:**
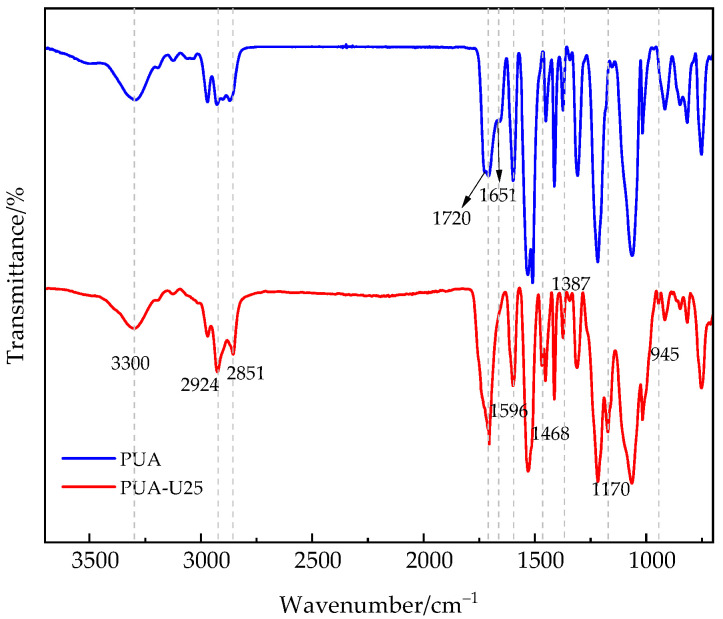
FTIR spectra of the PUA and PUA-U25 coating films.

The structure of the PUA coating film and urushiol-modified PUA(PUA-U25) was characterized by Fourier transform infrared (FTIR) as shown in Figure 2. Figure 2 shows a stretching vibration peak at 3300 cm^−1^, which is attributed to the hydroxyl group (-OH). The two strong absorption peaks at 2924 and 2851 cm^−1^ are attributed to the C-H stretching vibration of CH_3_- and CH_2_-groups, the alkyl bending vibration at 1468 cm^−1^, and the absorption peak at 945 cm^−1^ belongs to the non-conjugated double bond absorption of trimethylene in the urushiol structure [24,25]. The absorption bands of C-H bending vibration are located at 1468 cm^−1^ and 1387 cm^−1^. This revealed the presence of urushiol [26]. Additionally, there is a broad absorption peak of urushiol at 3200–3500 cm^−1^ [27], which disappears in the PUA-U25 spectrum, and the absorption peak of NCO at 2250–2300 cm^−1^ also disappears, indicating that the isocyanate group reacts with phenolic hydroxyl groups.

For comparison, a C=O absorption peak was found at 1651, 1720 cm^−1^ in the PUA spectrum, which is attributed to the reaction between the isocyanate group and the hydroxyl group in water to generate urea. However, this peak was not found in the PUA-U25 spectrum, which may be due to the reaction between the hydroxyl group of urushiol and isocyanate, rather than water. All these phenomena indicate that NCO reacts with phenolic hydroxyl groups.

**Figure 3:**
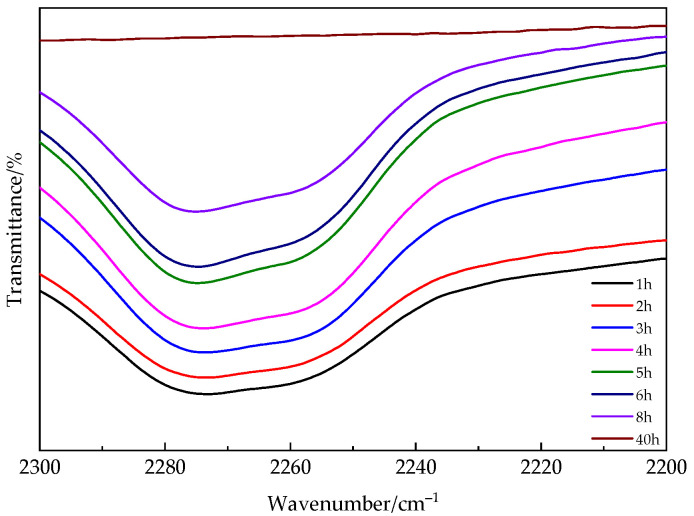
FTIR spectra of NCO changes over time in PUA-U25 coatings.

We also investigated the variation of the peak value of isocyanate functional groups over time through infrared spectroscopy. In Figure 3, the peak absorption of NCO functional groups decreases over time and nearly disappears after approximately 40 h. The requirement for an extended reaction time can be attributed to the internal diffusion process in the later stage of the curing reaction, which results in a low reaction rate.

#### 3.1.2. Mechanical Properties and Resistance of the Chemical Medium

Urushiol plays a crucial role in the resin curing process, affecting not only the curing rate but also the performance of the cured coating. Table 3 details the specific impact of different amounts of urushiol added on the mechanical properties of the film. All coatings show excellent adhesion to the substrate, largely due to the formation of urea bonds that combine with the matrix to form strengthened secondary hydrogen bonds. The hardness of the coating is directly influenced by the cross-linking density and the structure of the polymer skeleton. Experimental results indicate that as the content of urushiol gradually increases, the pencil hardness of the coating increases from 2H to 5H. Particularly, when the urushiol content is below 25% (w%), the film not only has moderate hardness but also good resistance to impact and flexibility. This balanced performance is attributed to the aromatic ring structures and flexible fatty chains present in the urushiol, as well as to the hard aromatic rings and flexible fatty rings formed by the reaction of isocyanate groups with the urushiol. However, when the content of urushiol exceeds 30% by weight, the overall performance of the coating begins to decline. This phenomenon is mainly due to the rigid aromatic ring structures in the urushiol, which, while enhancing pencil hardness, sacrifice the coating’s impact resistance and flexibility.

The original PUA film is renowned for its excellent chemical resistance, particularly in terms of tolerance to acids, alkalis, salts, and ethanol. In this study, chemical stability tests were conducted on both PUA and urushiol-modified PUA films. The observations are summarized in detail in Table 4. The test results indicate that the urushiol-modified PUA films maintained their chemical properties with little to no change after 7 days. However, an excessive addition of urushiol was found to significantly reduce the alkali resistance of the films, with the PUA-U30 film exhibiting wrinkling and bubbling after immersion, primarily due to the poor alkali resistance of urushiol itself. Additionally, Table 3 shows a decrease in the adhesion and impact resistance of the PUA-U30 film. An appropriate amount of urushiol modification does not compromise the chemical resistance of PUA but rather enhances its physical and mechanical properties. The excessive addition of urushiol is detrimental to the physical and chemical properties of the film. Therefore, the optimal modification ratio is achieved when the weight percentage of urushiol is 25%, making the PUA-U25 formulation the most favorable.

#### 3.1.3. Thermal Stability Analysis

**Figure 4:**
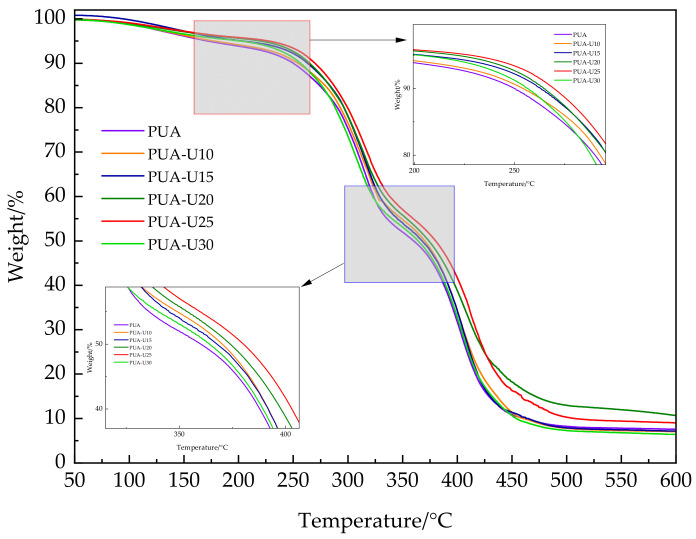
TG curves of PUA with variation in urushiol content.

Thermal stability is another critical factor that affects the performance of the coating film. Figure 4 presents thermogravimetric analysis data indicating that under a nitrogen atmosphere, both PUA and urushiol-modified PUA samples exhibited slight mass loss at temperatures below 200 °C, predominantly attributed to the volatilization of minute moisture content and residual solvents within the samples. The PUA material is composed of distinct hard and soft segment architectures, which undergo thermal degradation in two separate temperature intervals [28]. The first stage (250–330 °C) of weight loss is attributed to the cleavage of the urea bonds in the hard segments due to decomposition reactions. The second stage of weight loss, which occurs between 330 and 480 °C, is caused by the degradation of the soft segments. The modification with urushiol can increase the decomposition temperature, which may be due to the reaction of phenolic hydroxyl groups in urushiol with isocyanate groups, forming a highly cross-linking network structure that enhances the thermal stability of the film. When the proportion of urushiol exceeds 30%, there is a downward trend in the decomposition temperature of both stages, possibly due to phase separation caused by excessive addition, which is consistent with the mechanical performance and chemical resistance of the film. The thermogravimetric curve is consistent with the typical thermal degradation trend of typical polyurethane materials [29,30]. The results of the thermogravimetric test indicate that the urushiol-modified PUA has excellent heat resistance, aging characteristics, and stable high-temperature performance.

#### 3.1.4. Microscopic Morphology Analysis

**Figure 5:**
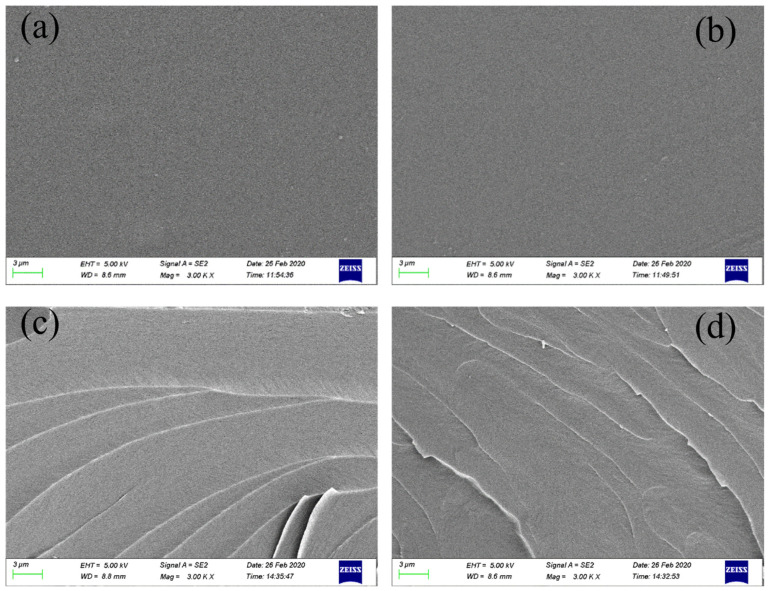
SEM images of the PUA surface (**a**), the PUA-U25 surface (**b**), the PUA cross-sectional morphology (**c**), the PUA-U25 cross-sectional morphology (**d**).

The surface and fracture surface morphology of the PUA coating film (Figure 5a) and PUA-U25 (Figure 5b) were observed using scanning electron microscopy (SEM). The surface morphology images reveal a smooth, uniform, and dense surface, with no “cave” phenomena or fractures observed. Cross-sectional SEM images indicate that the interior of the film is fully cured, with no porous structures or cracks, exhibiting a compact morphology, suggesting good compatibility between urushiol and PUA resin.

The high compatibility of urushiol with PUA resin is attributed to the chemical reaction between the phenolic hydroxyl groups and the isocyanate groups. This chemical bonding contributes to the formation of a highly transparent mixture without affecting the transparency and gloss of the paint film, consistent with the fact that urushiol does not affect the gloss as shown in Table 3.

Furthermore, the morphology of the fracture surface presents a typical brittle fracture pattern, clearly reflecting the brittleness of the polymer network. In summary, SEM image analysis has revealed the microstructure and integrity of the PUA and PUA-U25 films.

### 3.2. Analysis of Rheological Properties of Urushiol-Modified PUA

#### 3.2.1. Rheological Characterization: Isothermal Curing

Although the changes in FTIR spectra at different times mentioned above can provide an intuitive understanding of the cross-linking reaction process of the PUA-U25 system, quantitative analysis of the curing process is challenging. To overcome this limitation, rheokinetic methods were employed in this study to continuously describe the curing behavior of PUA and urushiol. All rheological experiments were conducted using the optimal PUA-U25 formulation.

**Figure 6:**
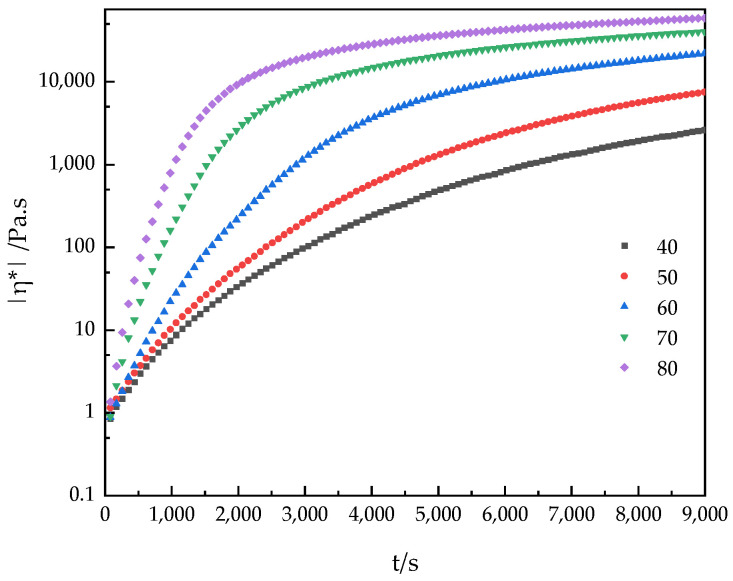
The time-dependent isothermal viscosity curve |η*| (color-coded mark) of the PUA-U25 system in the thermal range of 40–80 °C.

**Figure 7:**
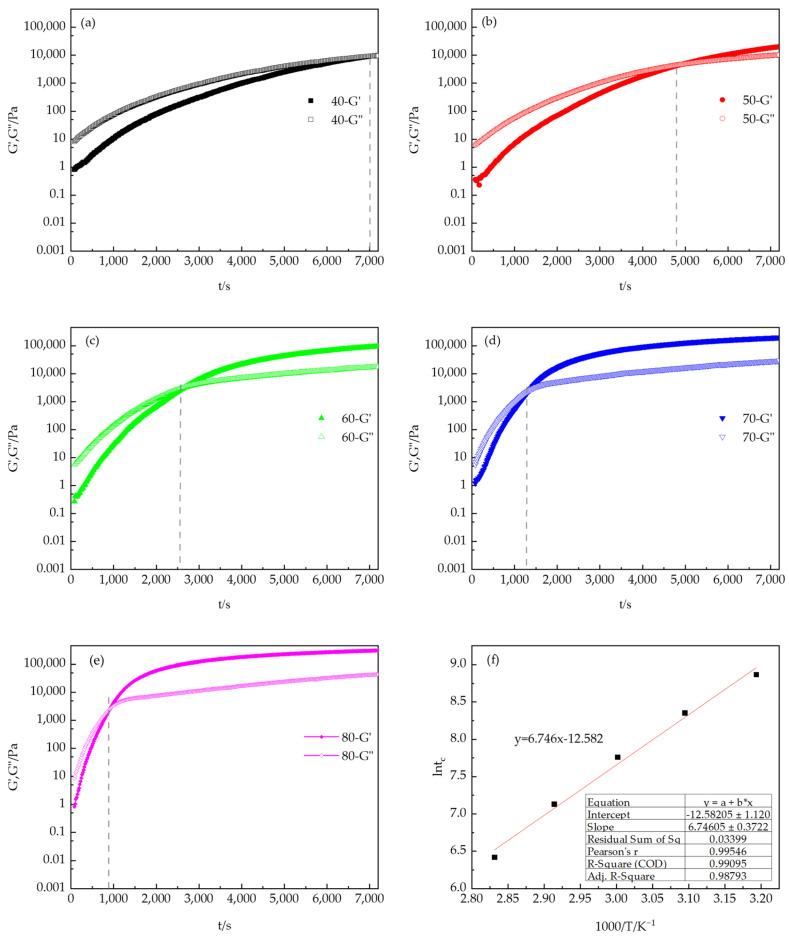
Variation of the dynamic moduli (G′ and G″, color-coded mark) with the reaction time of the PUA-U25 system during curing at different temperatures: (**a**–**e**) represent 40 °C to 80 °C, respectively, and (**f**) represents the Arrhenius expression of t_c_ as a reciprocal temperature function (the gel point at t_c_ was determined at the G′ and G″ crossover).

Viscosity control during the coating process is particularly important because viscosity varies not only with temperature and flow conditions but also over time. Therefore, studying the effects of temperature and time on viscosity changes during the reaction process can effectively control the curing reaction, optimize the construction process, and control the performance of the final product [31,32]. Based on the above reasons, the viscosity changes of the PUA-U25 system in the curing reaction process were studied by rotary rheometer. This experimental technique has proved its simplicity and versatility in reaction monitoring. Figure 6 illustrates the changes in viscosity data over time under isothermal conditions at a fixed frequency within the temperature range of 40–80 °C. The viscosity of the curing reaction to the gel point can be well estimated through experimental viscosity changes. Figure 6 demonstrates an exponential increase in viscosity over time, primarily due to the formation of a cross-linking network, which enhances the interactions between polymer molecules and restricts the movement of molecular chains. With increasing temperature, the curing reaction intensifies, and the viscosity of the system increases rapidly. When the reaction temperature is 80 °C, the viscosity of the system changes little after 60 min, indicating that the curing reaction is nearly complete. Figure 7 shows the variation curves of G′ and G″ over time at different curing temperatures at a frequency of 6.28 rad/s (1 Hz). The evaluation of the polymer network structure formed can be evaluated through dynamic rheological parameters including G′. As anticipated, the application of dynamic rheology for monitoring the curing process offers distinct advantages, with the technique being applicable for the entire duration of curing. Analysis of the time-scan curves identifies two primary stages in the curing process of the PUA-U25 system: the autocatalytic stage and the internal diffusion stage [33,34,35]. In the PUA-U25 system, the curing process lacks an induction period, with the initial stage primarily characterized by the viscous liquid properties of small molecule resins, this is G″ > G′. This indicates that the rapid increasing in molecular weight and intermolecular cross-linking led to a faster increase rate of G′ than G″. The intersection point of G′ and G″, known as the gel point, can be observed on all time-scan curves [36]. It marks the transition of the PUA-U25 system mixture from liquid to solid state. Finally, the system enters the internal diffusion stage, and the increase in viscosity gradually slows down. As the reaction reaches complete conversion, G′ and G″ slowly reach a plateau due to the consumption of isocyanates. In addition, it can be seen from the figure that the higher the temperature, the shorter the gel time, and the faster the reaction. The results indicate that increasing the temperature accelerates the reaction.

#### 3.2.2. Viscosity Profile

Observations in the study indicate that, as the reaction temperature increases, the time required for modulus crossover decreases, a phenomenon consistent with the expected behavior of thermal activation processes. The distinct linear trend displayed in Figure 7f further corroborates this. Due to the thermally active nature of the gelation process, the previous research literature [37,38] has used the Arrhenius equation to describe the activation energy (*E_α_*) of gelation [39,40]. The Arrhenius equation is expressed as:

(1) $$t_{c}=t_{c,0}e^{-\frac{E_{a}}{RT}}$$

where t_c,0_ is a constant, *T* represents the absolute temperature, and *R* is the universal gas constant. By analyzing the slope of the straight line in Figure 7f, we calculated the *E_α_* value to be 56.08 kJ/mol using Equation (1). It is important to note that this determined activation energy does not directly reflect the chemical kinetics controlling the cross-linking reaction, but instead provides an in-depth understanding of the network formation process and the thermal processes that govern it.

**Figure 8:**
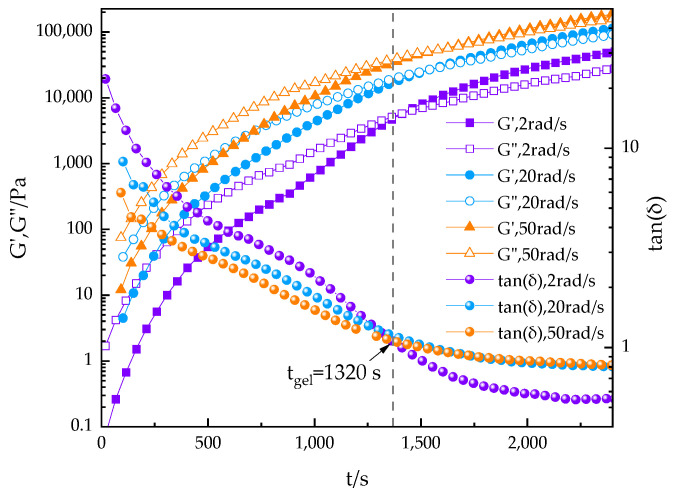
Viscoelastic properties (G′, G″, and tanδ) versus reaction time of the PUA-U25 system in multi-frequency mode at 70 °C. The gel point, t_gel_, is determined from the intersection point.

By recording rheological data at various frequencies, we determined the gelation time t_gel_ from the intersection of the loss tangent tanδ curves, as shown in Figure 8. This phenomenon indicates that at the gelation time t_gel_, the value of tanδ is independent of the angular frequency, suggesting that the cluster structure formed at this point has percolated macroscopically. Over time, the formation of the cross-linking polymer structure leads to an increase in G′ that exceeds that of G″, causing the tanδ value to gradually decrease. Before gelation time, tanδ decreases with increasing angular frequency ω, confirming the applicability of the Winter–Chambon criteria [41,42] to the PUA-U25 system.

From the intersection value on the horizontal axis of Figure 8, the tanδ value at the gel point can be determined. Using the appropriate formula, the relaxation exponent n can be further calculated.

(2) $$G^{\prime }\left(\omega \right)\sim G\prime \prime (\omega )\sim \omega^{n}$$

(3) $$\tan\left(\delta \right)=G\prime \prime (\omega )/G\prime (\omega )=tan(n\pi /2)$$

Here, *n* is the relaxation exponent, which depends on the molecular structure and details [43]. Through time-scan experiments at multiple oscillatory frequencies of 2, 20, and 50 rad/s, we determined the gel time t_gel_ at 70 °C. Since tanδ is independent of frequency at the gel point, the curve passes through a specific point, clearly defining the gel state. The experimental results show that the gel time (t_gel_) is 1320 s.

In all samples, the value of n_tan_ (approximately 0.55) is nearly consistent. Generally, the value of n can vary across the entire range of 0~1, depending on the stoichiometric ratio between cross-linking points and the molecular weight. When a critical gel with long chains is formed, the value of n is typically around 0.5 [44]. In the PUA-U25 system, this index is about 0.55, indicating that the critical gel chains are relatively long.

#### 3.2.3. Effect of Shear Frequency

**Figure 9:**
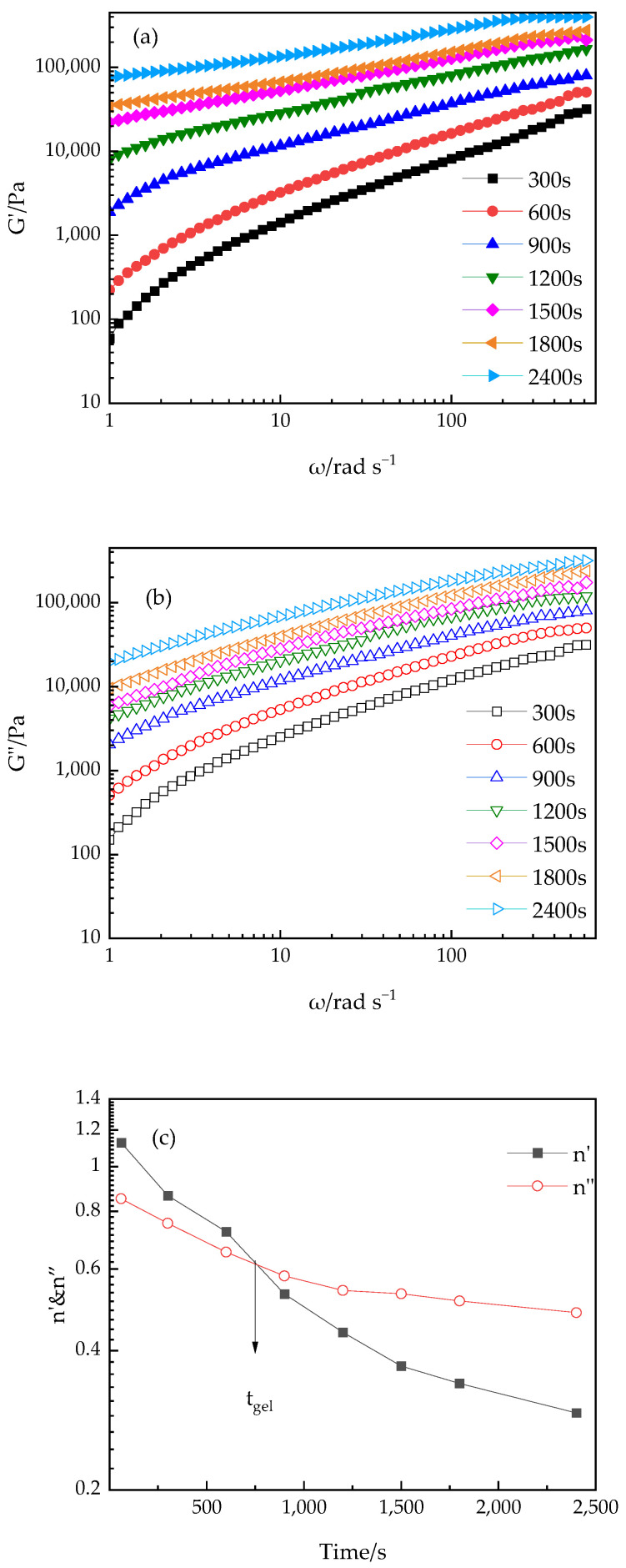
Variation of G′ as a function of angular frequency (**a**) of the PUA-U25 system; the change in G″ with angular frequency (**b**) of the PUA-U25 system; time dependence of exponents n′ and n′′ obtained from the data of G′ and G″ at 70 °C (**c**). The arrow indicates the gel time, t_gel_.

Under isothermal conditions, a study of heat-induced gelation of the PUA-U25 system was conducted across a wide frequency range. Figure 9a,b displays the frequency dependence of G′ and G″ at 70 °C at various time intervals. As the angular frequency ω and the gelation time increase, the values of G′ and G″ also increase. In the later stages of the gelation process, G′ and G″ become independent of frequency, reaching an equilibrium value (G_eq_) due to the formation of an elastic fractal gel.

The variation of G′ and G″ with angular frequency can be described by the power-law relationship of Equation (2), which is applicable throughout the entire frequency range. Exponents n′ and n″ are determined by the slopes of the G′ and G″ versus ω plots, and these exponent values are related to the gelation time. Figure 9c illustrates the time-dependent behavior of n′ and n″ at 70 °C. Both exponents decrease exponentially over time and converge at the gel point; that is, at t_gel_, n′ = n″ = 0.61. The experimental results reveal that the values of n_slope_ and n_tan_ are very close, confirming the correctness of the theoretical expectations. This phenomenon indicates that the estimated n-value remains stable under different temperature conditions. More importantly, the n is consistent with the value of 2/3 predicted by percolation theory [45], providing strong experimental support for the theoretical model.

#### 3.2.4. Reaction Kinetics by Rheometry: Non-Isothermal Curing

Isothermal curing rheology is an effective technology to evaluate the curing characteristics of thermosetting resins. It can measure the curing and gel time, and analyze the response of materials to temperature and frequency. However, in industrial production, thermosetting resins, including polyurethane, are typically prepared through a series of curing steps carried out in a specific sequence at different temperatures to precisely control the material’s performance. Therefore, to further investigate the curing behavior of the PUA-U25 system, this experiment conducts dynamic temperature scanning within a range of 40 °C to 180 °C, using various heating rates.

**Figure 10:**
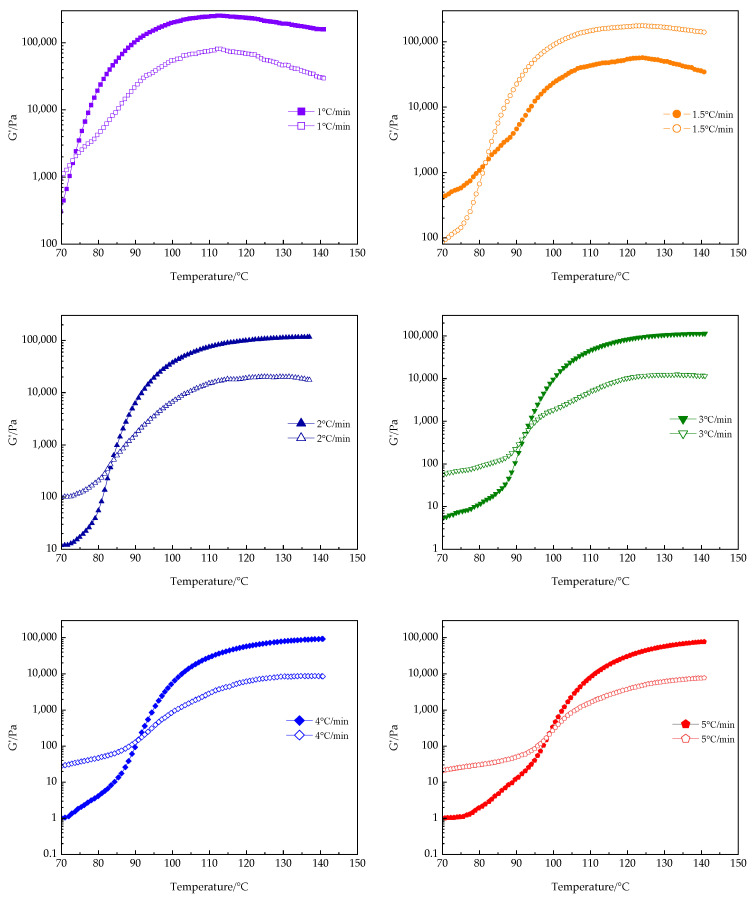
Storage modulus (G′) and loss modulus (G″) versus temperature at various heating rates of the PUA-U25 system.

The trends of the storage modulus (G′) and loss modulus (G″) at different heating rates are shown in Figure 10. At the initial stage of the curing reaction, G′ remains less than G″. As the temperature rises, the curing reaction accelerates, causing G′ to increase rapidly and eventually surpass G″, which signifies the formation of the cross-linking network and the gelation of the system. When G′ is greater than G″, the system primarily exhibits elastic properties, consistent with the progressive development of the isothermal cross-linking structure. Furthermore, the gelation time (t_gel_) decreases with an increase in the heating rate, and the gelation point shifts to a higher temperature, a phenomenon that is consistent with the observations of other researchers [46,47]. The decrease in gelation time is attributed to the rapid local cross-linking of the PUA-U25 system at high temperatures and fast heating rates, where the network structure inhibits the diffusion of unreacted small molecules. This is consistent with the reaction characteristics observed in isothermal curing processes, where higher curing temperatures lead to a shorter time to reach gelation.

**Figure 11:**
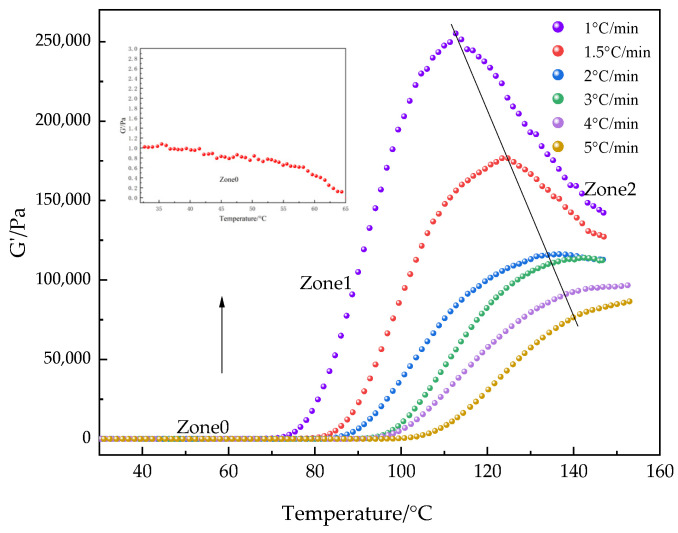
The non-isothermal rheological curve of the PUA-U25 system.

To describe the relationship between storage modulus and temperature, Figure 11 illustrates the whole process of how G′ changes with temperature. We can see that the entire process is divided into three regions. In Zone 0, there is an initial decrease in the storage modulus, while the rapid increase in the storage modulus in Zone 1 signifies the beginning of the curing process for thermosetting materials. The decrease in the storage modulus in Zone 2 indicates the completion of the curing process. Since the effect of temperature on the storage modulus is minor in the initial stage, the influence of temperature on the storage modulus can be neglected during this phase. In the final stage, after the curing process is completed, the increase in temperature at a low heating rate leads to a significant decrease in the storage modulus. This impact can be quantified by Equation (4), which defines the relationship between the increase in temperature and the decrease in the storage modulus [12,48,49], expressed as:

(4) $$k=\frac{∆G_{t}^{\prime }}{T_{t}-T_{0}}$$

Here, Δ*G*′(*t*) represents the decrease in the storage modulus due to the increase in temperature, *T*_*t*_ and *T*_0_ represent the temperature at the test time t and the temperature at the beginning of the reaction, respectively, and k is the slope of the storage modulus changing with temperature, which can be determined by linear fitting of the data.

Further, the transformation can be expressed as:

(5) $$\alpha =\frac{G_{test}^{\prime }\left(t\right)+k\left(T_{t}-T_{0}\right)-G_{0}^{\prime }}{G_{test}^{\prime }\left(\infty \right)+k{\left(T_{\infty }-T_{0}\right)}^{0}-G_{0}^{\prime }}$$

Here, *G*′_test_ (*t*) is the storage modulus at the curing process time t, and *G*′_test_ (∞) is the storage modulus at the end of the curing process. In this way, we can describe and predict changes in the storage modulus with temperature and time more accurately.

**Figure 12:**
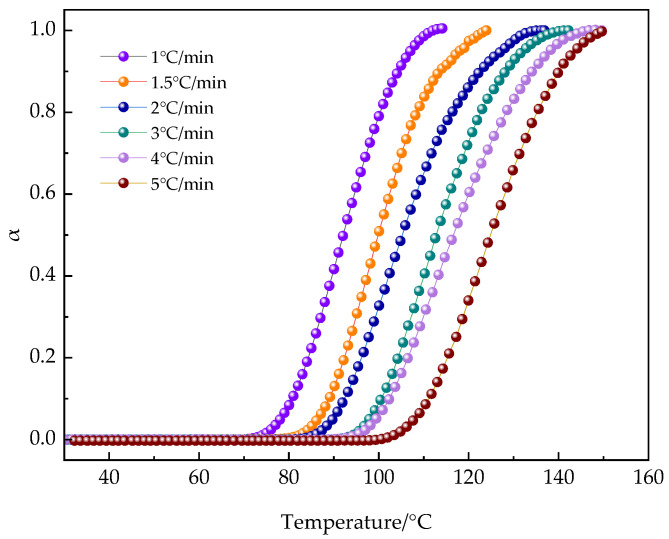
The law of conversion rate changes with temperature during the curing process of the PUA-U25 system.

Figure 12, representing Equation (5), illustrates the degree of conversion at various heating rates. It is evident that during the initial stages of curing, the increase in all α values is very gradual. After reaching a certain extent, the reaction is triggered, and the degree of curing increases rapidly, then gradually approaches a certain value. 

In the curing process, the conversion rate α versus temperature curves (Figure 12) all exhibit an S-shape, and shift to the right with increasing heating rate, consistent with the autocatalytic curing mechanism.

To describe the curing kinetics of thermosetting systems, the autocatalytic model from references [50,51,52,53] is commonly used to express the rate equation, which is specifically shown in Equation (7):

(6) $$\frac{d\alpha }{dt}=k\left(T\right)\alpha^{m}{\left(1-\alpha \right)}^{n}$$

where *α* is the degree of cure corresponding to the conversion rate dα/dt, k is the reaction rate constant that fluctuates with temperature, and *m*, *n* is the reaction order.

(7) $$k=Ae^{\frac{-E_{\alpha }}{RT}}$$

where A is the pre-exponential factor, and R is the gas constant, *Eα* is the activation energy.

**Figure 13:**
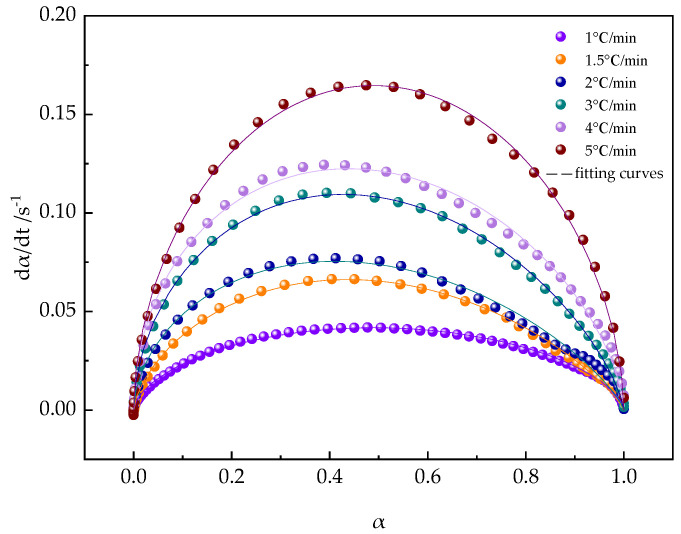
Non-isothermal autocatalytic simulation data and experimental data comparison of the PUA-U25 system.

According to Equation (6), the kinetic parameters k, m, and n were determined by fitting the experimental data with non-linear least squares fitting (NLSF), and the results are listed in Table 5. As expected, with the progress of the reaction, the value of k increases with increasing curing temperature, while the total reaction order (m + n) does not show significant changes. When comparing the predicted results with the experimental results shown in Figure 13, it was found that the two matched very well. This discovery indicates that rheological non-isothermal methods are effective not only for studying the mechanism of heterogeneous reactions but also that the autocatalytic reaction model is highly suitable for describing the progress of the non-isothermal curing reaction of the PUA-U25 system.

#### 3.2.5. Evaluation of the Activation Energy (*E_α_*)

The determination of *E_α_* is crucial in the study of the curing reaction kinetics. The equivalent transformation method of differential and integral equations provides the primary means for determining *E_α_* [54,55,56,57,58]. This paper uses the integral method proposed by Kissinger–Akahira–Sunose (KAS) to study the deviation of *E_α_*, thereby gaining a deeper understanding of the kinetic characteristics during the curing process of the material. The mathematical expression of the KAS method is shown in Equation (8):

(8) $$ln\frac{\beta }{T^{2}}=ln\frac{AR}{E_{\alpha }g\left(\alpha \right)}-\frac{E_{\alpha }}{RT}$$

*g*(α) is an integral form of the *f*(α).

(9) $$g\left(\alpha \right)=\int_{0}^{\alpha }\frac{d\alpha }{f\left(\alpha \right)}$$

**Figure 14:**
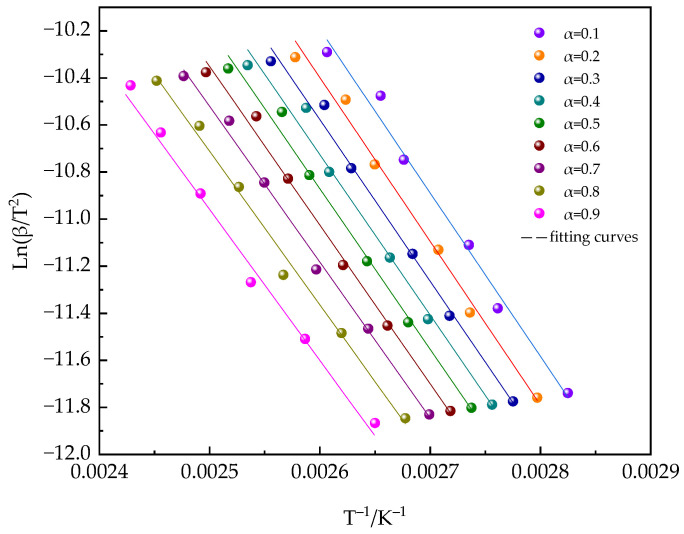
Ln (β/T^2^) versus 1/T of the PUA-U25 system.

**Figure 15:**
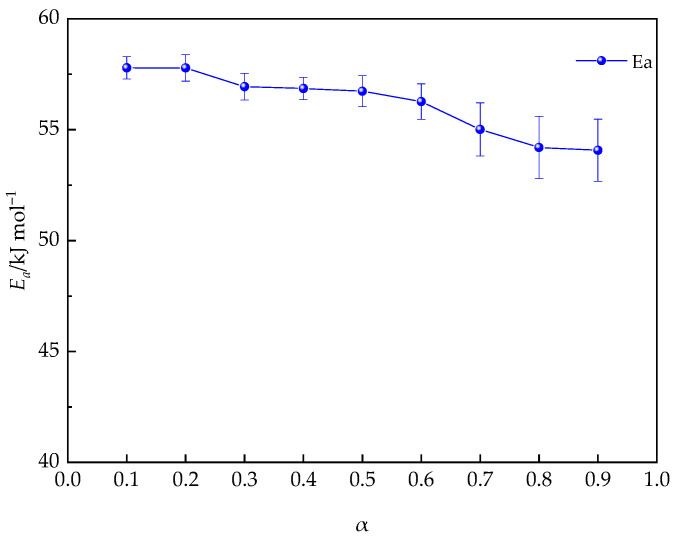
The evolution of *E_α_* with the conversion rate during the curing process of the PUA-U25 system.

In Figure 14, the KAS conversion curves for the PUA-U25 system at various values of *α* are presented. By analyzing the linear slope of the logarithmic of β/T^2^ against 1/T from Figure 14 and the corresponding calculated results, *E_α_* can be determined. Furthermore, Figure 15 illustrates the changing trend of *E_α_* vs. *α*. Within the range of 0.1 < α < 0.6, *E_α_* exhibits a high degree of stability, consistently measured at 57 kJ mol^−1^. The value obtained is consistent with the activation energy determined through isothermal calculations conducted in this research, and it aligns with the activation energies commonly reported in the literature for most polyurethane reactions [59,60]. When α surpasses 0.70, a significant drop in *E_α_* is observed, suggesting that diffusion control starts to become the predominant factor. Thus, in the later stages of the curing reaction, the further development of the reaction depends mainly on the short-range movement of adjacent groups, which is the key factor causing the rapid decrease in activation energy [61].

## 4. Conclusions

This study investigated the modification effects of urushiol as a cross-linking agent on commercial polyurethane acrylate (PUA), confirming that the urushiol-modified PUA can significantly enhance the cross-linking density of the resin, thereby improving the film formation properties, mechanical strength, and thermal stability. Rheological methods were employed to thoroughly analyze the isothermal and non-isothermal curing processes of the PUA-U25 system. Isothermal rheological experiments indicated that the gel time can be determined by the increase in the storage modulus G′ and its intersection with the loss modulus G″, and the gelation time significantly shortens as the curing temperature increases. By monitoring the changes in the storage modulus, non-isothermal rheokinetic analysis quantified the extent of reaction conversion and calculated the apparent activation energy using the Kissinger–Akahira–Sunose (KAS) method, providing new theoretical support for the curing kinetics. The research results not only validated the effectiveness of rheological testing but also offered an important reference model for the process optimization and further development of urushiol-based polyurethane.

## Figures and Tables

**Table 1:** The physicochemical parameters of PUA.

Physicochemical Parameter	Value
Appearance	Transparent liquid
Solid content (105 °C for 1 h)	70 ± 2%
Acid value	≤7
Viscosity	1500–2300 CPS
Isocyanate (NCO) value	7 ± 0.5%
Solvent	Butyl Acetate/Ethyl Acetate/ Propylene Glycol Methyl Ether Acetate

**Table 2:** Urushiol cross-linked PUA resins with varying urushiol content ratios.

Sample	PUA	PUA-U10	PUA-U15	PUA-U20	PUA-U25	PUA-U30
PUA (g)	100	100	100	100	100	100
Urushiol (g)	0	10	15	20	25	30

**Table 3:** Mechanical properties of various samples.

Type	Gloss/GU	Pencil Hardness	Adhesion	Bending Test/mm	Frontal Impact/kg·cm
PUA	200	2H	0	<2	50
PUA-U10	200	3H	0	<2	50
PUA-U15	200	4H	<1	<2	50
PUA-U20	200	4H	<1	<2	50
PUA-U25	200	5H	<1	<2	50
PUA-U30	200	5H	2	<3	40

**Table 4:** Chemical resistance of various samples.

Type	NaCl, 10% (*w*/*w*)	H_2_SO_4_, 10% (*w*/*w*)	NaOH, 10% (*w*/*w*)	C_2_H_5_OH, 95% (*v*/*v*)
PUA	+	+	+	+
PUA-U10	+	+	+	+
PUA-U15	+	+	+	+
PUA-U20	+	+	+	+
PUA-U25	+	+	+	+
PUA-U30	+	+	−	+

+: Unaffected; −: wrinkling or blistering.

**Table 5:** Autocatalytic model reaction orders (m, n) and the rate constant k in the non-isothermal curing process.

Heat Rate °C/min	k	m	n	R2
1	0.0954	0.5844	0.6071	0.9975
1.5	0.1802	0.6392	0.8220	0.9966
2	0.1863	0.5481	0.7875	0.9936
3	0.2592	0.5401	0.7236	0.9999
4	0.2638	0.4896	0.6311	0.9999
5	0.3534	0.5389	0.5635	0.9999

## Data Availability

The data underlying this study are available in the published article; further inquiries can be directed to the corresponding author/s.

## References

[1] Moon H., Park J.E., Cho W., Jeon J., Wie J.J. (2023). Curing kinetics and structure-property relationship of moisture-cured one-component polyurethane adhesives. Eur. Polym. J..

[2] Ghosh B., Gogoi S., Thakur S., Karak N. (2016). Bio-based waterborne polyurethane/carbon dot nanocomposite as a surface coating material. Prog. Org. Coat..

[3] Kozakiewicz J., Rokicki G., Przybylski J., Sylwestrzak K., Parzuchowski P.G., Tomczyk K.M. (2011). Studies on the effect of curing conditions on the curing rate and mechanical properties of moisture-cured poly(urethane-urea) elastomers containing oligocarbonate segments. Polimery.

[4] Song X., Lin Y., Zhu C., Huang J., Bai X., Zhang H., Hu H., Li G. (2022). Biooxazolidines-enabled improvement of monocomponent polyurethane coatings. Macromol. Mater. Eng..

[5] Nomura Y., Sato A., Sato S., Mori H., Endo T. (2007). Synthesis of novel moisture-curable polyurethanes end-capped with trialkoxysilane and their application to one-component adhesives. J. Polym. Sci. Pol. Chem..

[6] John G., Nagarajan S., Vemula P.K., Silverman J.R., Pillai C.K.S. (2019). Natural monomers: A mine for functional and sustainable materials—Occurrence, chemical modification and polymerization. Prog. Polym. Sci..

[7] Zhang Y., Li T.T., Shiu B.C., Lin J.H., Lou C.W. (2022). Multifunctional sodium alginate@ urushiol fiber with targeted antibacterial, acid corrosion resistance and flame retardant properties for personal protection based on wet spinning. Appl. Surf. Sci..

[8] Fang Y., Yan L., Liu H. (2020). Facile preparation of hydrophobic melamine sponges using naturally derived urushiol for efficient oil/water separation. ACS Appl. Polym..

[9] Xia J., Lin J., Xu Y., Chen Q. (2011). On the UV-induced polymeric behavior of Chinese lacquer. ACS Appl. Mater. Inter..

[10] Yang W., Liang B., Tan W., He X., Lv J., Xiao H., Zeng K., Hu J., Yang G. (2020). Rheological study on the cure kinetics of dicyanimidazole resin. Thermochim. Acta.

[11] Domínguez J.C., Madsen B. (2014). Chemorheological study of a polyfurfuryl alcohol resin system-pre-gel curing stage. Ind. Crops. Prod..

[12] He W., He L., Ma Z., Guo Y. (2016). Using Rheometry to study the curing kinetics of glycidyl azide polymer spherical propellant by non-isothermal method. Rheol. Acta.

[13] Malkin A.Y., Kulichikhin S.G. (1996). Rheokinetics: Rheological Transformations in Synthesis and Reactions of Oligomers and Polymers.

[14] Lucio B., De La Fuente J.L. (2014). Rheological cure characterization of an advanced functional polyurethane. Thermochim. Acta.

[15] Zhang Y., Fang R., Xue H., Xia J., Lin Q. (2022). Investigation of DSC curing kinetic model fitting and rheological behavior of urushiol/IPDI System. Thermochim. Acta.

[16] Zhang Y., Fang R., Xue H., Ye Y., Lin J., Lin Q., Xia J. (2023). Study on rheological behaviors and rheokinetics of urushiol/MDI resin system during curing process. Thermochim. Acta.

[17] Xu H., Lu Z., Zhang G. (2012). Synthesis and properties of thermosetting resin based on urushiol. RSC Adv..

[18] Latimer G.W. (2023). Official Methods of Analysis of AOAC International.

[19] (1988). Test Method for Gloss of Plastic Mirror Surface.

[20] (2006). Paints and Varnishes–Determination of Film Hardness by Pencil Test.

[21] (2021). Paints and Varnishes–Cross-Cut Test.

[22] (2007). Paints and Varnishes–Bend Test (Cylindrical Mandrel).

[23] (1993). Paints and Varnishes–Determination of Impact Resistance of Film.

[24] Yin F., Lin J., Mao X., Deng Y., Chen Q. (2013). Preparation and characterization of bamboo fibers coated with Titanium urushiol and its composite materials with polypropylene. Int. Polym. Proc..

[25] Lee H., Han H., Kim D., Lee B., Cho J.H., Lee Y., Lee S.-S., Lim J.A. (2021). Mixed urushiol and laccol compositions in natural lacquers: Convenient evaluation method and its effect on the physicochemical properties of lacquer coatings. Prog. Org. Coat..

[26] Lu R., Honda T., Ishimura T., Miyakoshi T. (2005). Study of a naturally drying lacquer hybridized with organic silane. Polym. J..

[27] Xia J., Xu Y., Lin J., Hu B. (2008). UV-induced polymerization of urushiol without photoinitiator. Prog. Org. Coat..

[28] Lu M.G., Lee J.Y., Shim M.J., Kim S.W. (2002). Thermal degradation of film cast from aqueous polyurethane dispersions. J. Appl. Polym. Sci..

[29] Jiang Y., Yuan K., Li S., Zhou Y. (2006). FTIR spectroscopy and thermal analysis of polyurethanes. Spectrosc. Spectr. Anal..

[30] Jin X., Guo N., You Z., Wang L., Wen Y., Tan Y. (2020). Rheological properties and micro-characteristics of polyurethane composite modified asphalt. Constr. Build. Mater..

[31] Zheng S.X., Chen H.S. (2023). Correlations of rheological methods to coatings’ performance. Prog. Org. Coat..

[32] Patel A., Maiorana A., Yue L., Gross R.A., Manas-Zloczower I. (2016). Curing kinetics of biobased epoxies for tailored applications. Macromolecules.

[33] Halley P.J., Mackay M.E. (1996). Chemorheology of thermosets—An overview. Polym. Eng. Sci..

[34] Yang H., Liu Z., Yang Y., Zheng Q. (2012). Rheologic studies on chemical cross-linking kinetics for LDPE. Chin. J. Polym. Sci..

[35] Li J., Si Z., Shang K., Feng Y., Wang S., Li S. (2023). Kinetic and chemorheological evaluation on the crosslinking process of peroxide-initiated low-density polyethylene. Polymer.

[36] Thomas R., Yumei D., Yuelong H., Le Y., Moldenaers P., Weimin Y., Czigany T., Thomas S. (2008). Miscibility, morphology, thermal, and mechanical properties of a DGEBA based epoxy resin toughened with a liquid rubber. Polymer.

[37] Chiou B.S., English R.J., Khan S.A. (1996). Rheology and photo-cross-linking of Thiol-Ene Polymers. Macromolecules.

[38] Raghavan S.R., Chen L.A., McDowell C., Khan S.A., Hwang R., White S. (1996). Rheological study of crosslinking and gelation in chlorobutyl elastomer systems. Polymer.

[39] Weng L., Chen X., Chen W. (2007). Rheological characterization of in situ crosslinkable hydrogels formulated from oxidized dextran and N -carboxyethyl chitosan. Biomacromolecules.

[40] Madbouly S.A., Xia Y., Kessler M.R. (2013). Rheological behavior of environmentally friendly castor oil-based waterborne polyurethane dispersions. Macromolecules.

[41] Chambon F., Winter H.H. (1987). Linear viscoelasticity at the gel point of a crosslinking PDMS with imbalanced stoichiometry. J. Rheol..

[42] Winter H.H. (1987). Can the gel point of a cross-linking polymer be detected by the G′-G″ crossover?. Polym. Eng. Sci..

[43] Tokita M., Nishinari K. (2009). Gels: Structures, Properties, and Functions.

[44] Henning Winter H., Izuka A., De Rosa M.E. (1994). Experimental observation of the molecular weight dependence of the critical exponents for the rheology near the gel point. Polym. Gels Netw..

[45] Takenaka M., Kobayashi T., Hashimoto T., Takahashi M. (2002). Time evolution of dynamic shear moduli in a physical gelation process of 1, 3:2, 4-Bis-O-(p-Methylbenzylidene)-D-sorbitol in polystyrene melt: Critical exponent and gel strength. Phys. Rev. E.

[46] Park J.O., Yoon B.J., Srinivasarao M. (2011). Effect of chemical structure on the crosslinking behavior of bismaleimides: Rheological study. J. Non-Newton. Fluid Mech..

[47] Lucio B., Fuente J.L. (2016). Non-isothermal DSC and rheological curing of ferrocene-functionalized, hydroxyl-terminated polybutadiene polyurethane. React. Funct. Polym..

[48] He W., Tao B., Yang Z., Yang G., Guo X., Liu P.J., Yan Q.L. (2019). Mussel-inspired polydopamine-directed crystal growth of core-shell n-Al@PDA@CuO metastable intermixed composites. Chem. Eng. J..

[49] Ma Z., Qi L., He W., He L. (2019). A novel approach on the study of cure kinetics for rheological isothermal and non-isothermal methods. Compos. Part B Eng..

[50] He W., Guo J.H., Cao C.K., Liu X.K., Lv J.Y., Chen S.W., Liu P.J., Yan Q.L. (2018). Catalytic reactivity of graphene oxide stabilized transition metal complexes of triaminoguanidine on thermolysis of RDX. J. Phys. Chem. C.

[51] Domínguez J.C., Alonso M.V., Oliet M., Rojo E., Rodríguez F. (2010). Kinetic study of a phenolic-novolac resin curing process by rheological and DSC analysis. Thermochim. Acta.

[52] Šesták J., Berggren G. (1971). Study of the kinetics of the mechanism of solid-state reactions at increasing temperatures. Thermochim. Acta.

[53] Malkin A.Y., Beghishev V.P., Keapin I.A., Andrianova Z.S. (1984). General treatment of polymer crystallization kinetics-Part 2. The kinetics of nonisothermal crystallization. Polym. Eng. Sci..

[54] Flynn J.H., Wall L.A. (1966). General treatment of the thermogravimetry of polymers. J. Res. Natl. Bur. Stan. Sect. A..

[55] Ozawa T. (2006). A new method of analyzing thermogravimetric data. Bull. Chem. Soc. Jpn..

[56] Vyazovkin S. (1997). Advanced isoconversional method. J. Therm. Anal..

[57] Vyazovkin S. (2001). Modification of the integral isoconversional method to account for variation in the activation energy. J. Comput. Chem..

[58] Akahira T., Sunose T. (1971). Method of determining activation deterioration constant of electrical insulating materials. Res. Rep. Chiba. Inst. Technol..

[59] Stanko M., Stommel M. (2018). Kinetic prediction of fast curing polyurethane resins by model-free isoconversional methods. Polymers.

[60] Milanese A.C., Cioffi M.O.H., Voorwald H.J.C., Shigue C.Y. (2011). Cure kinetic of castor oil-based polyurethane. J. Appl. Polym. Sci..

[61] Liang T., Li C., Pan D., Song G., Mai X., Naik N., Vupputuri S., Guo Z. (2020). Rheological non-isothermal mechanistic investigation on the curing of glycidyl azide polymer with solid nanofillers. React. Funct. Polym..

